# The Assessment of Health-Related Quality of Life in Patients With Chronic Liver Disease: A Single-Center Study

**DOI:** 10.7759/cureus.10727

**Published:** 2020-09-30

**Authors:** Ravi R Pradhan, Brindeswari Kafle Bhandari, Rahul Pathak, Sagar Poudyal, Shahbaz Anees, Sashi Sharma, Prem Khadga

**Affiliations:** 1 Internal Medicine, Tribhuvan University Institute of Medicine, Kathmandu, NPL; 2 Gastroenterology, Tribhuvan University Institute of Medicine, Kathmandu, NPL; 3 Gastroenterology, Institute of Medicine, Tribhuvan University Teaching Hospital, Kathmandu, NPL; 4 Gastroenterology, KIST Medical College, Kathmandu, NPL

**Keywords:** health related quality of life, chronic liver disease, nepali version sf-36

## Abstract

Aim

The aim of this study was to investigate the factors affecting health-related quality of life (HRQOL) in Nepalese patients with chronic liver disease (CLD).

Methods

In this study, HRQOL was measured with validated Nepali versions of the short-form 36 (SF-36) survey. Socioeconomic factors, etiology, laboratory parameters, disease severity, and self-rated health perceptions on HRQOL were recorded for analysis.

Results

Sixty CLD patients were enrolled in the study. The following HRQOL scores were obtained: physical functioning (PF) (34.4 ±26.7), role limitation due to physical health (RLPH) (7.5 ±17.8), role limitation due to emotional problems (RLEP) (27.7 ±38.2), energy or fatigue (E/F) (38.5 ±21.5), emotional well-being (EWB) (57.7 ±22.8), social functioning (SF) (55.2 ±23.5), pain (44.8 ±30.3), and general health (GH) (38.2 ±17). Employed status and higher annual family income had a positive impact on HRQOL. Ascites and abnormal upper gastrointestinal endoscopic findings were associated with poor health status perceptions. More severe disease (higher Child-Pugh class) was associated with lower HRQOL scores. A significant negative correlation between the model for end-stage liver disease (MELD) score and HRQOL domains was observed (p: <0.05). Age, gender, religion, education, and duration of the diagnosis of CLD had no effect on HRQOL of CLD patients.

Conclusion

HRQOL in patients with CLD was lower than that in the general population. Unemployed status, low annual family income, ascites, abnormal upper gastrointestinal endoscopic findings, and higher Child-Pugh class and MELD scores were important factors that adversely affected HRQOL.

## Introduction

Chronic liver disease (CLD) is one of the major causes of morbidity and mortality worldwide. Globally, CLD has affected 844 million people, with an annual mortality rate of two million [1]. Liver cirrhosis is the 11th most common cause of death globally [2]. Additionally, it has been estimated that liver cirrhosis is within the top 20 causes of disability-adjusted life years and years of life lost [2]. Patients with CLD may suffer from anxiety, depression, and other emotional problems. Furthermore, complications like ascites, hepatic encephalopathy, variceal bleeding, and spontaneous bacterial peritonitis may have a negative impact on their health-related quality of life (HRQOL) [3]. Also, non-life-threatening symptoms such as fatigue, muscle and joint pain, pruritus, loss of appetite, and digestive problems can hugely decrease their quality of life and well-being [4,5]. The multidimensional concept of HRQOL includes different domains relating to emotional, mental, social, and physical problems in the context of the disease and its management [6].

There have been many studies conducted on the socio-demographic profile and etiology of CLD in Nepal; however, in literature reviews, no such study has been conducted to assess the HRQOL of CLD patients. The main objective of this study was to investigate the factors affecting HRQOL in patients with CLD using a validated Nepali version of the short-form 36 (SF-36) survey.

## Materials and methods

We conducted an observational, cross-sectional study at the Institute of Medicine (IOM), Kathmandu, Nepal over a period of six months from 8th February 2018 to 7th August 2018. The study protocol was approved by the Institutional Review Committee (IRC) of the IOM. A total of 60 CLD patients were included in the study. CLD was diagnosed by the treating physician or hepatologist based on clinical, biochemical, serological, and imaging evidence of portal hypertension and/or liver dysfunction with a history of illness of more than six months [7]. CLD patients of age <16 years, and those with cognitive impairment, stroke, chronic obstructive pulmonary disease, heart diseases, and people living with HIV/AIDS (PLHA) were excluded as these could potentially affect their HRQOL and act as confounding factors. We collected socio-demographic, clinical, and laboratory details of the patients using a structured questionnaire. The disease severity was measured using the Child-Pugh stage (stage A, B, and C) [8]. Ultrasonography (USG) of the abdomen was performed to grade ascites [9]. Upper gastrointestinal endoscopy (UGIE) was performed for each patient to know about persisting esophageal/gastric varices (EV/GV), and/or portal hypertensive gastropathy (PHG).

We validated the Nepali language version of SF-36, which had already been published, by distributing it among 40 patients [10]. These patients were not included in the present study. The validated Nepali version of SF-36 was then used to assess the HRQOL of CLD patients. Educated patients were asked to fill the form by themselves. For those patients who were illiterate, we read out the questions for them and their responses were noted. Prior written informed consent was obtained from all patients after explaining the nature and purpose of the study. SF-36 has eight domains and 36 items. Physical functioning (PF), role limitations due to physical health (RLPH), role limitations due to emotional problems (RLEP), energy/fatigue (E/F), emotional well-being (EWB), social functioning (SF), pain, and general health (GH) are the eight domains of SF-36. The raw score was calculated using the mean score of the items within each domain. The calculated raw scores were converted into a 0-100 scale using a RAND 36 score calculator. A higher score denoted better HRQOL.

Statistical analysis

We used EpiData v 3.1 for data entry and SPSS Statistics v 16 (IBM, Armonk, NY) for the analysis of data. The Shapiro-Wilk test was employed to check the normality of distribution of the collected data, and we found that our data were not normally distributed. Continuous variables were expressed as mean ±standard deviation (SD), and categorical variables were expressed as frequency and percentages. Bivariate relationships between different domains of HRQOL and socio-demographic factors or clinical parameters were analyzed using the Mann-Whitney U test and the Kruskal-Wallis test. Spearman’s correlation coefficient was used to assess the correlation between the model for end-stage liver disease (MELD) score and different domains of HRQOL. A p-value of <0.05 was considered to be statistically significant.

## Results

Socio-demographic characteristics of the patients

The mean age of the patients was 52.5 ±9.8 years. Among the 60 patients, 60% were male, 70% were Hindus, 75% were unemployed, 55% were from the Hilly region of Nepal, 60% belonged to the Janjati caste (based on the traditional caste system), 40% were illiterate, and 91.7% were married. A majority of the patients (70%) had an annual family income of less than 500,000 Nepalese rupees (NPR) ($1 is equivalent to around 123 NPR). The mean duration of the diagnosis of CLD among the patients was 17 months (SD=27) (Table 1).

**Table 1:** Socio-demographic characteristics of the patients NPR: Nepalese rupee

Variables		Frequency	Percentage
Gender	Male	36	60.0
	Female	24	40.0
Religion	Hindu	42	70.0
	Buddhist	16	26.7
	Muslim	2	3.3
Employment	Employed	15	25.0
	Unemployed	45	75.0
Residential region	Terai	20	33.3
	Hilly	33	55.0
	Himalayan	7	11.7
Ethnicity	Brahmin	7	11.7
	Chhetri	9	15.0
	Madhesi	5	8.3
	Dalit	3	5.0
	Janjati	36	60.0
Educational level	Illiterate	24	40.0
	Primary	21	35.0
	Secondary	11	18.3
	Higher secondary or university	4	6.7
Marital status	Unmarried	1	1.7
	Married	55	91.7
	Divorced	2	3.3
	Widowed	2	3.3
Family income per year, NPR	<1 lakh	15	25.0
	1-5 lakh	27	45.0
	5-10 lakh	11	18.3
	>10 lakh	7	11.7
Alcohol consumption	Yes	49	81.7
	No	11	18.3
Smoking	Yes	45	75.0
	No	15	25.0

Etiology of CLD, Child-Pugh stage, ascites grade, and endoscopic findings among the patients

The three most common etiology of CLD were found to be alcohol (75%), viral (13.3%), and cryptogenic (5%) (Table 2).

**Table 2:** Etiology of chronic liver disease NAFLD: non-alcoholic fatty liver disease

Etiology	Frequency	Percentage
Alcohol	45	75.0
Hepatitis B	6	10.0
Hepatitis C	2	3.3
Autoimmune hepatitis	1	1.7
NAFLD	2	3.3
Cryptogenic	3	5.0
Cardiac cirrhosis	1	1.7

Out of 60 patients, 30 (50%) were in Child-Pugh stage C and 52 (86.7%) had ascites. The majority of the patients (86.7%) had either EV or GV or PHG. Among the 52 patients with ascites, 28 (46.7%) had grade 3 ascites (Table 3).

**Table 3:** Child-Pugh stage, ascites grade, and endoscopic findings among the patients UGIE: upper gastrointestinal endoscopy; EV: esophageal varices; GV: gastric varices; PHG: portal hypertensive gastropathy

Parameters		Frequency	Percentage
Child-Pugh stage	A	4	6.7
	B	26	43.3
	C	30	50.0
Ascites	Absent	8	13.3
	Present	52	86.7
Ascites grade	Grade 1	11	18.3
	Grade 2	13	21.7
	Grade 3	28	46.7
UGIE	Normal	8	13.3
	EV/GV/PHG	52	86.7

Health-related quality of life and correlation with different parameters

The HRQOL score was lowest for the domain RLPH (7.5 ±17.8) and highest for EWB (57.7 ±22.8) (Table 4).

**Table 4:** Health-related quality of life score for different domains

Domains	Mean	Standard deviation
Physical functioning	34.4	26.7
Role limitation due to physical health	7.5	17.8
Role limitation due to emotional problems	27.7	38.2
Energy or fatigue	38.5	21.5
Emotional well-being	57.7	22.8
Social functioning	55.2	23.5
Pain	44.8	30.3
General health	38.2	17

Males had higher score for PF (31.46 vs 29.06, p=.602), E/F (31.51 vs 28.98, p=.58), and pain (31.31 vs 29.29, p=.661) compared with females. Similarly, scores for RLEP and GH were higher in females compared with males (31.75 vs 29.67, p=.612 and 34.98 vs 27.51, p=.102). Muslims had higher scores for RLEP (36.75), E/F (39.25), EWB (45), SF (35), and pain (40) compared with Hindus and Buddhists; however, the difference was statistically insignificant. Unemployed individuals had significantly lower scores for PF (27.47 vs 39.6, p=.019), E/F (27.32 vs 40.03, p=.014), and pain (27.41 vs 39.77, p=.017) compared with employed patients. Similarly, patients from Terai and Hilly regions had significantly lower scores for PF (25.78 vs 29.67 vs 47.97, p=.014), RLPH (26.72 vs 30.38 vs 41.86, p=.023), SF (32.65 vs 26.47 vs 43.63, p=.049) and pain (23.5 vs 32.62 vs 40.5, p=.049) compared with those from Himalayan region. We discovered that education did not have any effect on HRQOL of CLD patients. We also noticed that patients of the Madhesi ethnic group had significantly lower scores in the GH domain compared with Brahmins, Chhetris, Dalits, and Janjatis (16.9 vs 39.14 vs 43.72 vs 40.5 vs 26.57, p=.012). Patients with an annual family income of more than 10 lakhs NPR had a significantly higher score in EWB (46.58 vs 21.30 vs 33.19 vs 25.00, p=.010) and SF (48.75 vs 28.40 vs 28.17 vs 26.45) domains compared with those with an annual family income of less than 10 lakhs (Table 5).

**Table 5:** Health-related quality of life and correlation with socio-demographic profile PF: physical functioning; RLPH: role limitations due to physical health; RLEP: role limitations due to emotional problems; E/F: energy/fatigue; EWB: emotional well-being; SF: social functioning; GH: general health; NPR: Nepalese rupee

Variables		PF (mean rank)	RLPH (mean rank)	RLEP (mean rank)	E/F (mean rank)	EWB (mean rank)	SF (mean rank)	Pain (mean rank)	GH (mean rank)
Gender	Male	31.46	30.68	29.67	31.51	30.56	30.22	31.31	27.51
	Female	29.06	30.23	31.75	28.98	30.42	30.92	29.29	34.98
	P-value	.602	.892	.612	.58	.976	.878	.661	.102
Religion	Hindu	31.17	31.74	29.64	30.60	29.48	30.02	30.27	32.07
	Buddhist	29.56	28.06	31.97	29.16	31.38	31.19	29.91	26.19
	Muslim	24.00	24.00	36.75	39.25	45.00	35.00	40.00	32.00
	P-value	.824	.462	.744	.740	.456	.908	.733	.508
Occupation	Employed	39.60	32.33	30.40	40.03	36.83	32.20	39.77	31.47
	Unemployed	27.47	29.89	30.53	27.32	28.39	29.93	27.41	30.18
	P-value	.019	.514	.977	.014	.104	.659	.017	.803
Residential region	Terai	25.78	26.72	29.68	26.25	30.48	32.65	23.50	27.78
	Hilly	29.67	30.38	30.56	30.24	29.18	26.47	32.62	32.00
	Himalayan	47.93	41.86	32.57	43.86	36.79	43.36	40.50	31.21
	P-value	.014	.023	.914	.070	.577	.049	.049	.685
Education	Illiterate	27.81	30.27	30.73	27.44	24.75	26.69	24.75	31.08
	Primary level	36.21	35.40	30.57	41.55	37.86	37.69	39.21	29.55
	Secondary level	24.05	24.00	24.18	18.32	24.77	22.68	24.64	30.68
	Higher secondary or university level	34.38	24.00	46.12	24.38	42.12	37.12	35.38	31.50
	P-value	.209	.065	.119	.002	.025	.053	.024	.991
Marital status	Unmarried	49.50	24.00	55.50	49.50	27.50	37.50	21.00	56.00
	Married	29.59	30.55	30.17	29.51	30.51	30.28	30.99	29.55
	Divorced	31.50	39.00	28.75	35.50	24.75	20.50	21.25	35.25
	Widow or widower	45.00	24.00	28.75	43.25	37.50	43.00	31.00	39.25
	P-value	.435	.633	.448	.456	.903	.593	.824	.391
Ethnicity	Brahmin	33.71	31.79	35.71	33.93	44.50	35.79	39.43	39.14
	Chhetri	36.89	40.78	35.17	35.00	35.50	38.50	35.50	43.72
	Madhesi	32.30	24.00	25.50	26.60	29.20	32.40	26.00	16.90
	Dalit	27.33	35.67	18.00	36.83	13.50	15.50	38.67	40.50
	Janjati	28.29	28.15	30.06	28.72	28.12	28.46	27.46	26.57
	P-value	.703	.056	.396	.754	.066	.245	.322	.012
Income per year, NPR	<1 lakh	28.97	30.73	29.67	23.03	21.30	28.40	25.67	29.00
	1-5 lakh	28.46	26.96	26.37	32.48	33.19	28.17	32.96	29.46
	5-10 lakh	31.59	31.50	30.86	31.50	25.00	26.45	29.18	29.36
	>10 lakh	36.58	39.08	45.58	33.50	46.58	48.75	29.00	36.08
	P-value	.740	.170	.049	.335	.010	.041	.611	.835

Patients with CLD secondary to autoimmune hepatitis and non-alcoholic fatty liver disease (NAFLD) had significantly lower scores for RLPH (24) compared to patients with CLD of other etiologies. We found that patients with Child-Pugh stage C had lower scores in all domains of HRQOL, and their scores in domains SF (22), E/F (22.08), and pain (26.90) were significantly lower compared with patients in stages A and B. The domains PF, E/F, pain, and GH scores were significantly lower in patients with ascites compared with those without ascites. Patients with grade 3 ascites and abnormal findings in UGIE (EV/GV/PHG) had lower scores in all domains of HRQOL (Table 6).

**Table 6:** Health-related quality of life and correlation with etiology of CLD, Child-Pugh stage, ascites, and UGIE PF: physical functioning; RLPH: role limitations due to physical health; RLEP: role limitations due to emotional problems; E/F: energy/fatigue; EWB: emotional well-being; SF: social functioning; GH: general health; NAFLD: non-alcoholic fatty liver disease; UGIE: upper gastrointestinal endoscopy; EV: esophageal varices; GV: gastric varices; PHG: portal hypertensive gastropathy; CLD: chronic liver disease

Variables		PF (mean rank)	RLPH (mean rank)	RLEP (mean rank)	E/F (mean rank)	EWB (mean rank)	SF (mean rank)	Pain (mean rank)	GH (mean rank)
Etiology	Alcohol	28.82	27.78	28.90	30.18	28.90	27.98	29.80	29.13
	Hepatitis B	35.67	39.00	26.67	36.58	32.17	32.17	35.25	33.67
	Hepatitis C	57.00	54.00	44.00	50.75	51.75	43.00	44.75	47.75
	Autoimmune	3.50	24.00	18.00	2.00	8.00	1.50	4.50	13.00
	NAFLD	17.00	24.00	39.50	19.50	35.50	48.50	4.50	39.25
	Cryptogenic	38.67	35.67	43.00	17.50	31.50	44.83	40.50	39.00
	Cardiac cirrhosis	51.50	59.00	55.50	57.50	59.50	59.00	53.00	13.00
	P-value	.080	.006	.226	.088	.236	.064	.082	.452
Child-Pugh stage	A	44.25	39.00	30.60	49.00	34.75	40.25	54.25	44.38
	B	38.19	32.65	31.48	37.37	34.37	34.17	31.00	31.29
	C	22.00	27.50	23.38	22.08	26.58	26.02	26.90	27.97
	P-value	.001	.116	.624	<0.001	.219	.106	.013	.195
Ascites	Absent	46.38	35.25	33.88	47.94	38.06	41.56	45.81	47.50
	Present	28.06	29.77	29.98	27.82	29.34	28.80	28.14	27.88
	P-value	.006	.251	.510	.002	.187	.051	.008	.003
Ascites grade	Grade 1	38.09	31.27	27.82	38.32	35.86	28.50	32.82	36.09
	Grade 2	24.73	27.46	28.19	24.62	30.00	28.12	26.23	25.81
	Grade 3	22.77	24.18	25.20	22.73	21.20	24.96	24.14	23.05
	P-value	.015	.149	.749	.013	.015	.724	.271	.049
UGIE	Normal	38.56	35.25	34.75	40.50	38.25	36.69	33.31	44.50
	EV/GV/PHG	29.26	29.77	29.85	28.96	29.31	29.55	30.07	28.35
	P-value	.160	.251	.407	.081	.176	.275	.624	.014

A significant negative correlation was observed between MELD scores and all domains of HRQOL except the domain of RLEP. Age and duration of diagnosis of CLD did not show any significant correlation with the domains of HRQOL (Table 7).

**Table 7:** Health-related quality of life and correlation with MELD score, age, and duration of the diagnosis of CLD MELD: model for end-stage liver disease; CLD: chronic liver disease; PF: physical functioning; RLPH: role limitations due to physical health; RLEP: role limitations due to emotional problems; E/F: energy/fatigue; EWB: emotional well-being; SF: social functioning; GH: general health

Variables		PF	RLPH	RLEP	E/F	EWB	SF	Pain	GH
MELD score	Correlation coefficient	-.482	-.351	-.150	-.596	-.291	-.378	-.423	-.259
	P-value	<0.001	.006	.252	<0.001	.024	.003	.001	.046
Age	Correlation coefficient	.084	.174	.142	.130	.068	.083	.090	.110
	P-value	.522	.184	.280	.321	.606	.528	.493	.404
Duration of the diagnosis of CLD in months	Correlation coefficient	.023	.023	.026	.092	.183	.146	.029	.086
	P-value	.861	.860	.841	.487	.161	.266	.825	.513

## Discussion

HRQOL is an important parameter to assess the disease progression and efficacy of management in patients with CLD. The chronic nature of CLD has an adverse effect on HRQOL of the patients. In the present study, we aimed to evaluate HRQOL in patients with CLD at a tertiary care center in Nepal, and we found that the patients had low self-perceptions of HRQOL in all the domains compared with the general Nepalese population [11]. HRQOL score was highest for the domain EWB, suggesting that the Nepalese are emotionally strong (Tables 4, 8).

**Table 8:** Health-related quality of life scores for different domains among the general population SD: standard deviation

Domains	Gender	Mean	SD	P-value
Physical functioning	Male	80.22	14.15	<0.001
	Female	75.26	11.01	
Role limitation due to physical health	Male	85.99	26.78	.811
	Female	86.33	12.65	
Role limitation due to emotional problem	Male	73.07	14.16	.249
	Female	71.90	13.84	
Energy or fatigue	Male	63.37	16.73	<0.001
	Female	58.61	17.81	
Emotional well-being	Male	84.83	19.46	.153
	Female	82.9	17.98	
Social functioning	Male	83.00	7.27	.256
	Female	82.42	6.91	
Pain	Male	80.97	27.05	.005
	Female	75.64	25.50	
General health	Male	67.16	17.91	.728
	Female	66.71	17.96	

We discovered that CLD due to chronic alcohol consumption (75%) was the most common cause of the disease. This finding is consistent with the study by Bhattarai et al. [12] conducted in the central part of Nepal.

Although employed individuals in this study scored better in every domain of HRQOL than those who were unemployed, the results were statistically significant only for the PF, E/F, and pain domains. This finding is consistent with that of Sobhonslidsuk et al. [13] and Marchesini et al. [4] who showed significant improvement in HRQOL with employment. The unemployed status may lead to financial burden and keep the patients away from appropriate treatment, leading to the deterioration of the liver disease [13].

It is interesting to note that the geographical locations of the people affected their HRQOL scores. Patients from the Terai and Hilly regions scored lower in every domain of HRQOL compared with those from the Himalayan region. Similarly, patients of the Madhesi ethnic group had a poor overall perception of GH. This is the first study associating HRQOL scores with geographical locations and ethnicity in patients with CLD in Nepal.

Income appears to be a novel predictor of HRQOL. Patients with an annual family income of more than 10 lakhs NPR had higher scores in all domains of HRQOL and the domains SF and pain showed statistically significant results regarding the same. This is in accordance with the study by Sobhonslidsuk et al. [13]. Patients with higher income can easily fulfill their needs and afford better treatment. Additionally, good financial security leads to a better sense of well-being and self-esteem, and less worry about the future, all of which result in better HRQOL.

There are other socio-demographic factors that seem to affect HRQOL as reported by other studies [13], which were not found to be a significant predictor of HRQOL in our study. We did not observe any effect of gender, religion, and education on the HRQOL of CLD patients. Häuser et al. [14] also found that gender and social class have no effect on HRQOL of CLD patients.

We could not find any significant correlation regarding age and duration of diagnosis of CLD with HRQOL (Table 7). This finding is comparable to similar findings by Souza et al. [15] and Häuser et al. [14]. During the early phase of CLD, most of the patients are asymptomatic or have mild symptoms, and they may not perceive a significant effect on their HRQOL. As the disease progresses with time, patients may feel a decline in their HRQOL. In our study, the mean duration of diagnosis of CLD was 17 months; it is highly probable that patients had not yet experienced the symptoms that are expected to occur with advanced stages and longer duration of the disease course. This may explain the absence of the effect of the duration of diagnosis of CLD on HRQOL in our study.

We did not discover any significant difference in HRQOL domains based on the etiology of CLD except for the domain of RLPH. CLD secondary to autoimmune hepatitis and NAFLD had significantly lower scores for RLPH compared to CLD of other etiologies (Table 6). This finding is in contrast with the original study by Ray et al. [16], in which patients with CLD of viral etiology had the lowest HRQOL score. Since the number of respondents from these etiologies in our study was very small (autoimmune hepatitis: 1.7%; NAFLD: 3.3%), bigger sample size is required before we can draw a definite conclusion regarding this.

We found that the presence of ascites and abnormal UGIE findings (i.e. EV/GV/PHG) had a negative impact on HRQOL. Patients with ascites had significantly lower scores for the domains PF, E/F, pain, and GH compared to those without ascites. We also noticed that as the grade of ascites increased, it negatively affected all the domains of HRQOL (T). These findings are similar to a study by Ray et al. [16]. We found that when the liver disease became severe, as indicated by a higher Child-Pugh class, HRQOL also became more impaired. This finding is similar to other studies [15,16]. However, it is in contrast with a study by Häuser et al. [14], who observed that the Child-Pugh class had no effect on HRQOL of CLD patients. HRQOL is negatively affected by ascites and hepatic encephalopathy, both being components of the Child-Pugh score. This might explain the negative correlation of Child-Pugh class with the HRQOL score. We found a significant negative correlation between the MELD score and almost all domains of HRQOL of patients with CLD. This is in contrast with the findings of Souza et al. [15].

Our study has some limitations. Since our study was cross-sectional, we could not describe how the progression of the disease affected HRQOL. As the study was conducted at a single tertiary healthcare center in Nepal, the results should not be generalized and may not be applicable to patients seen in primary care centers. While the majority of our patients self-responded to the HRQOL questionnaire (i.e., Nepali version of SF-36), illiterate patients had to have the questionnaire read out for them by an investigator, and their responses were noted by the investigator. Reporting biases may have crept in for such cases.

## Conclusions

Using a validated Nepali version of SF-36, we found that the patients with CLD had low overall HRQOL scores compared with the general Nepalese population. HRQOL scores were lowest for the domain RLPH and highest for the domain EWB. Unemployed status, low annual family income, ascites, abnormal UGIE findings, and higher Child-Pugh class and MELD scores are important factors that have a negative impact on HRQOL. HRQOL of CLD patients was not affected by age, gender, religion, education, and duration of the diagnosis of CLD. We recommend that a further large-scale, multi-center study be carried out in patients with CLD in Nepal to assess the overall HRQOL of Nepalese CLD patients.
